# Deep learning for microbiome-informed precision nutrition

**DOI:** 10.1093/nsr/nwaf148

**Published:** 2025-04-16

**Authors:** Yang-Yu Liu

**Affiliations:** Channing Division of Network Medicine, Department of Medicine, Brigham and Women's Hospital, Harvard Medical School, USA

Nutrition science, over the past several centuries, has evolved from focusing on the isolation and synthesis of individual nutrients to a more sophisticated understanding of the intricate biological impacts of food and dietary patterns. Particularly in the last century, varied dietary patterns have spurred extensive scientific inquiry linked to chronic diseases. For instance, studies have consistently affirmed the efficacy of the Mediterranean diet in mitigating cardiovascular disease risk and reducing overall mortality [1].

Nowadays, a multi-omics approach is frequently invoked to formulate strategies for precision nutrition. The National Institutes of Health defines Precision Nutrition as an encompassing framework that integrates diverse elements, including genetics, dietary habits, circadian rhythms, health status, and socioeconomic and psychosocial factors, alongside food environments, physical activity, and microbiome composition. This paradigm acknowledges the intrinsic individual differences among humans, suggesting that when, why, and how we eat are as critical as what we eat.

The objectives of precision nutrition research are manifold. They range from enhancing the precision of dietary and nutritional status measurements (utilizing tools like biomarkers or smartphone applications) to a more nuanced comprehension of the biological mechanisms underpinning diet. This extends to refining dietary recommendations at the population level, encompassing specifics such as types of fats and carbohydrates, and daily intake limits for sodium, sugar, and saturated fatty acids. Additionally, a significant aim is to elevate the precision of individual dietary advice, leveraging artificial intelligence for personalized diet prescriptions, thereby advancing personalized nutrition. Despite criticisms concerning the evidence supporting personalized nutrition and the excessive promotion of certain companies, the transition from generic healthy guidelines to personalized dietary recommendations appears both natural and inevitable [2]. This is underscored by the highly personalized biological and lifestyle determinants that cause diverse metabolic responses to specific foods and nutrients in individuals.

The human gut microbiome plays a central role in digesting food, synthesizing vitamins, regulating the immune system, and producing bioactive metabolites like short-chain fatty acids. It also influences how the human body absorbs, stores, and metabolizes nutrients. Since the gut microbiome composition varies significantly between individuals, the same diet can have different metabolic and health effects in different people. Consequently, gut microbiome-informed precision nutrition is an emerging approach that tailors dietary recommendations based on an individual's unique gut microbial composition and function. Rather than offering one-size-fits-all guidelines, it uses data from microbiome sequencing, metabolomics, and host-microbe interactions to predict how someone will respond to specific foods or nutrients.

**Figure 1.**
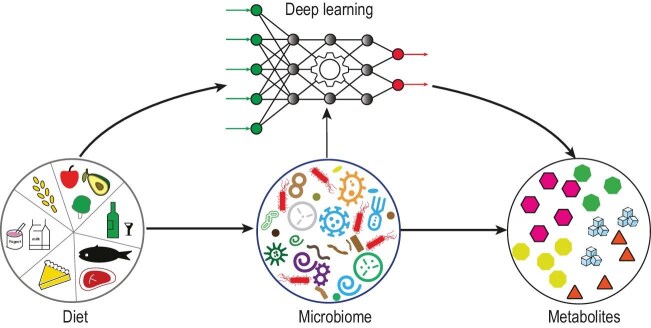
Deep learning facilitates microbiome-informed precision nutrition. By analyzing the gut microbiome, we can predict how an individual will respond to specific foods and dietary interventions, leading to more effective and personalized dietary recommendations. This microbiome-informed precision nutrition approach can be facilitated with deep learning techniques and metabolomics.

Deep learning, a subset of machine learning, is adept at identifying patterns and making predictions from large data sets, making it an invaluable tool in precision nutrition, especially microbiome-informed precision nutrition (see Fig. 1). In this Perspective, we delve into several deep-learning methods (cNODE, mNODE, McMLP, and METRIC) related to microbiome-informed precision nutrition. Each method is examined for its theoretical underpinnings, operational mechanisms, and practical applications. Among those methods, McMLP and METRIC are directly related to precision nutrition. For pedagogy purposes, we will introduce cNODE and mNODE as precursors of McMLP and METRIC.

The deep learning method cNODE (compositional Neural Ordinary Differential Equation) aims to predict microbial composition from the species assemblage of a microbial community [3]. The cNODE code and tutorial are available at https://github.com/michel-mata/cNODE.jl. Mathematically speaking, cNODE implicitly learns a map or a nonlinear function $\varphi $ that connects the binary vector ${\boldsymbol{z}}$ (representing the species presence/absence pattern in the sample) to the community composition vector ${\boldsymbol{p}}$, i.e. $\varphi :{\boldsymbol{z}} \to {\boldsymbol{p}}$. To ensure this map $\varphi $ exists and the problem is mathematically well defined, we need to make a few assumptions, i.e. universality [4], equilibrium [5], no true multi-stability [6]. While these assumptions are unlikely to be strictly met in reality, successful microbial composition predictions serve as *a posteriori* validation of these assumptions.

To learn the function $\varphi $, cNODE leverages Neural Ordinary Differential Equations (NODE), a relatively new neural network architecture [7]. The key idea of NODE is to integrate concepts from ordinary differential equations (ODEs) into neural network design. Instead of defining the neural network as a series of discrete layers that transform input data into an output, NODE defines a continuous flow of information through the network, allowing for more flexible and expressive modeling of complex data.

Based on NODE, we developed cNODE, which satisfies two conditions: (1) the predicted vector must be compositional; (2) the predicted abundance of any absent species must be zero. The architecture of cNODE's output layer is uniquely tailored to meet these conditions. We have rigorously tested cNODE against simulated data from a classical population dynamics model in community ecology and subsequently applied it to predict microbial compositions across diverse environments, including the ocean and soil microbiome, *Drosophila melanogaster* gut microbiome, and the human gut and oral microbiome.

The merit of cNODE lies not in the prediction of compositions when both presence/absence patterns and compositions are concurrently known for most microbiome datasets, but rather in performing thought experiments of species invasion or removal and predicting the community response. Indeed, we have shown that using a well-trained cNODE, we can perform thought experiments of species removal, and hence identify keystone species from complex microbial communities in a purely data-driven fashion, without using any ecological model or invoking any technical or ethical concerns [8].

Building on the success of cNODE, we developed mNODE (Metabolomic profile predictor using Neural Ordinary Differential Equations) [9]. The mNODE code can be found at https://github.com/wt1005203/mNODE. The primary objective of mNODE is to predict the metabolomic profile based on the microbial composition of a microbial community. Given the greater complexity and cost associated with metabolomics compared to metagenomics, there is a strong incentive to create computational methods capable of predicting metabolic profiles from microbial compositions. Additionally, such a method could enhance our understanding of the interactions between microorganisms and their metabolites through conducting thought experiments.

A significant distinction between cNODE and mNODE is that, in the case of cNODE, the species collection vector ${\boldsymbol{z}}$ and the microbial composition vector ${\boldsymbol{p}}$ share the same dimension, while in the case of mNODE, the microbial composition vector ${\boldsymbol{p}}$ and the metabolite concentration vector ${\boldsymbol{q}}$ typically do not share the same dimension. Consequently, the original NODE framework

is not applicable. Our approach involves transforming the input microbial composition vector ${\boldsymbol{p}}$ into a latent vector of dimension ${N}_{\mathrm{h}}$. This latent vector serves as the input for a NODE, which subsequently transforms the output (which is another latent vector of dimension ${N}_{\mathrm{h}}$) into the metabolomic profile. Essentially, we ‘sandwich’ the NODE module between two fully connected layers. The resulting mNODE architecture is highly versatile. It has the potential to incorporate various types of input data, such as dietary intake, to improve the prediction of metabolomic profiles.

To validate mNODE, we generated synthetic data using the Microbial Consumer-Resource model, which intricately simulates microbial dynamics through nutrient competition and metabolic cross-feeding. mNODE outperformed alternative methods in prediction accuracy across three distinct evaluation metrics. Enhancement of mNODE's predictive power was observed when inputs were expanded to include nutrient supply rates alongside microbial composition. Subsequent application to real datasets from four distinct studies corroborated mNODE's superior performance when benchmarked against the same three metrics.

A well-trained mNODE can help us conduct thought experiments to study the microbe-metabolite interactions. For example, we can perturb the relative abundance of species-*i* (by $\Delta {x}_i$) and measure how the predicted concentration for metabolite-$\alpha $ changes (by $\Delta {y}_\alpha $). The sign of susceptibility of metabolite-$\alpha $ to species-*i* is ${s}_{i\alpha } = \Delta {y}_\alpha /\Delta {x}_i$, which can be used to reflect the interaction between metabolite-$\alpha $ and species-*i*. Presumably, if ${s}_{i\alpha } > 0$ (or $< 0$), it may imply species-*i* can produce (or consume) metabolite-$\alpha $, respectively. We have applied the susceptibility analysis to a real dataset related to the fecal microbiome and metabolome samples of patients with IBD. We identified several interactions that agree with genomic evidence. For example, *Bacteroides vulgatus* contains the bile salt hydrolase (bsh) gene, which is responsible for the deconjugation of conjugated primary bile acids to primary bile acids cholate and chenodeoxycholate. Therefore, the susceptibility analysis based on a well-trained mNODE could be quite useful to explore microbe-metabolite interactions.

Achieving personalized nutrition hinges on the development of predictive models that integrate diverse individual data types to accurately forecast metabolomic profiles in response to dietary changes [10]. Most of the existing models are constrained to traditional machine learning methods such as Random Forest and Gradient-Boosting Regressor [11]. We recently developed McMLP (**M**etabolic response predictor using **c**oupled **M**ulti**l**ayer **P**erceptrons) [12], a deep learning method that predicts metabolite response to dietary intervention from the baseline gut microbial composition and metabolite concentrations and the dietary intervention strategy itself. The McMLP code is available at https://github.com/wt1005203/McMLP.

Inspired by the success of mNODE, one may naïvely think that mNODE can easily predict post-intervention metabolite response. It turns out that this assumption is overly optimistic. mNODE and other deep learning models, such as Multilyer Perceptron (MLP), struggled with this task when applied directly (i.e. in a one-step fashion). The challenge stems from the inherent differences in the objectives: mNODE is adept at analyzing the current microbial composition of a microbial community to infer its current metabolomic profiles, whereas the goal here is to predict post-intervention metabolite concentrations from baseline gut microbial compositions and metabolite concentrations.

McMLP is based on a two-step strategy: (1) predict the endpoint microbial composition via a classical deep learning model, i.e. MLP; (2) incorporate this predicted microbial composition into another MLP to predict the endpoint metabolite concentrations. The utilization of two coupled MLPs yields McMLP.

The validation of McMLP using synthetic data generated by the Microbial Consumer-Resource model indicated its superior performance over existing predictive models (e.g. RF and GBR) across multiple evaluation metrics. Incorporating baseline metabolomic profiles as input further enhanced all predictive models, particularly McMLP. Following rigorous validation with simulated data, we applied McMLP to real data from six dietary intervention studies, five of which were controlled feeding trials. McMLP consistently produces the best performance across all datasets, particularly when incorporating baseline metabolite concentrations as inputs.

Employing the susceptibility analysis, similar to that used in the mNODE study, we can examine the complex food-microbe-metabolite relationship. For example, when analyzing data from the avocado intervention study, we constructed the avocado-microbe-butyrate tripartite graph, which highlighted *Faecalibacterium prausnitzii* as a strong avocado-consuming and butyrate-producing species, corroborating earlier research. This suggests that a well-trained McMLP model could be instrumental in unveiling complex food-microbe-metabolite interactions, thus propelling forward personalized dietary recommendations for precision nutrition.

Enhancing the accuracy of dietary and nutritional status measurements is a central goal of precision nutrition. However, accurately capturing dietary intake in large-scale cohort studies is a significant challenge. Researchers often rely on self-reported instruments developed in nutritional epidemiology—such as the food frequency questionnaires, 24-hour recalls, and diet records. While widely used, these tools are prone to measurement errors, which can lead to inaccurate estimates of individual nutrient profiles. To address this issue, we developed **METRIC** (**M**icrobiom**e**-based Nu**t**rient P**r**of**i**le **C**orrector) [13], a deep learning model that leverages gut microbial composition data to correct random errors in nutrient profiles derived from self-reported dietary assessments, including 24-hour recalls and diet records. The METRIC code is available at https://github.com/wt1005203/METRIC.

Our initial aim was to infer the true nutrient profile using the assessed profile and the corresponding gut microbiome data. Ideally, this would involve training a machine learning model—such as a MLP or mNODE—using assessed nutrient profiles and microbial compositions as input, and ground-truth nutrient profiles (derived from the actual dietary intake data) as output. However, this approach is not feasible in practice, as ground-truth dietary intake data are rarely available. Inspired by the Noise2Noise [14] model developed in computer vision, we hypothesized that it is possible to correct random errors in assessed nutrient profiles without requiring ‘clean’ or ground-truth nutrient profile data. Specifically, METRIC focuses on correcting random errors with zero mean, rather than systematic biases, which would require access to true dietary intake data for effective correction.

METRIC's architecture consists of an input layer, three hidden layers (each with 256 units), and an output layer. A distinctive feature of METRIC is its skip connection, which adds the input (i.e. the corrupted nutrient profile) directly to the output of the neural network. Similar to its use in Noise2Noise, the skip connection helps constrain the model's predictions, ensuring they do not deviate excessively from the observed data while still enabling meaningful correction. To train METRIC, we simulated corrupted nutrient profiles by adding random noise to the assessed nutrient profiles. The model was trained to recover the original assessed profiles using the corrupted profiles and microbiome compositions as inputs. This training strategy prevents METRIC from merely replicating the input and instead compels the model to learn to remove noise. Importantly, no ground-truth nutrient profiles were used during training. In essence, METRIC functions as a general-purpose denoiser that learns to remove random noise from nutrient profile data. Once trained, METRIC can generate corrected nutrient profiles that are closer to the true profiles by reducing random measurement error—even in the absence of ground-truth data.

We demonstrated that METRIC performs exceptionally well in reducing simulated random errors, particularly for nutrients that are metabolized by gut microbes, using both synthetic datasets and three real-world datasets. Notably, METRIC also shows strong correction performance even without microbiome input, underscoring the robustness of the approach.

In conclusion, the application of deep learning techniques holds significant promise for microbiome-informed personalized nutrition, a key objective within the scope of precision nutrition. Further investigation and development in this research area are warranted.
